# A potential association between immunosenescence and high COVID-19 related mortality among elderly patients with cardiovascular diseases

**DOI:** 10.1186/s12979-021-00234-z

**Published:** 2021-06-01

**Authors:** Yuanyuan Wang, Shu-Chao Pang, Ying Yang

**Affiliations:** 1 Department of Cardiology, Hangzhou Xiacheng Hospital of Integrated Traditional Chinese and Western Medicine, Hangzhou, 310004 Zhejiang China; 2 The First Teaching Hospital of Tianjin University of Traditional Chinese Medicine, Tianjin, China; 3 Department of Cardiology, SirRunRunShaw Hospital, College of Medicine, Zhejiang University, No.3 Qingchun East Road, Hangzhou, 310016 Zhejiang China

**Keywords:** Immunosenescence, Elderly patients, Cardiovascular disease, COVID-19

## Abstract

Elderly patients with cardiovascular diseases account for a large proportion of Corona virus Disease 2019(COVID-19)related deaths. COVID-19, as a new coronavirus, mainly targets the patient’s lung triggering a cascade of innate and adaptive immune responses in the host. The principal causes of death among COVID-19 patients, especially elderly subjects with cardiovascular diseases, are acute respiratory distress syndrome(ARDS), multiple organ dysfunction syndrome (MODS), and microvascular thrombosis. All prompted by an excessive uncontrolled systemic inflammatory response. Immunosenescence, characterized by systemic and chronic inflammation as well as innate/adaptive immune imbalance, presents both in the elderly and cardiovascular patients. COVID-19 infection further aggravates the existing inflammatory process and lymphocyte depletion leading to uncontrollable systemic inflammatory responses, which is the primary cause of death. Based on the higher mortality, this study attempts to elucidate the pathophysiological mechanisms of COVID-19 in elderly subjects with cardiovascular diseases as well as the cause of the high mortality result from COVID-19.

## Background

COVID-19 is a pneumonia caused by SARS-CoV-2 infection. SARS-CoV-2, as a novel coronavirus, that has not been found in human before, which can make a strong strike to human immune system. Although the most patients with infection have a good prognosis, death from respiratory and circulatory failure due to COVID-19 is more likely in the elderly and those with chronic underlying diseases, especially in elderly patients with cardiovascular disease (CVD). This paper intends to elaborate the pathogenesis of high mortality of elderly CVD patients caused by COVID-19 from the perspective of immunosenescence.

## Advanced age and associated cardiovascular disease are independent risk factors of high mortality in COVID-19 patients

Previous studies have shown higher mortality rates among COVID-19 patients with CVD. For example, research involving 191 COVID-19 patients from Wuhan JinYinTan hospital and Wuhan Pulmonary Hospital has indicated a positive correlation between age and in-hospital mortality [1]. Two other studies on a total of 541 and 83 COVID-19 patients also showed a higher mortality rate among CVD patients (22.2 % vs. 9.8 % for the first study with 541 subjects) compared to the total or non-CVD population mortality rates [2, 3]. Additionally, another study with a total of 996 COVID-19 patients further indicated that hypertension is independently associated with all-cause mortality in elderly COVID-19 subjects [4]. Autopsies performed on 80 COVID-19 infected deceased patients (Germany) showed that all subjects belonged to an age range of 52 to 96 years (mean 79.2 years, median 82.4 years) and had underlying comorbidities, with conditions of the cardiovascular system being the first risk factors of mortality [5]. This connection between chronic diseases and COVID-19 related mortality among elderly patients was further supported by a Chinese study on 100 COVID-19 deaths, establishing a clear relationship,especially with cardiovascular diseases as well as diabetes [6]. It can be seen that old age and combined cardiovascular diseases are independent risk factors for high mortality of COVID-19 patients [7, 8], and the specific reasons are worth further discussion.

## Uncontrolled systemic inflammatory response and the multi-organ microvascular endothelial damage caused by it is the pathological basis of death in COVID-19 patients

Basic research has found that,It has been demonstrated that SARS-CoV-2 accesses the lungs through the ACE2 receptor, a most abundantly expressed receptor in the type II lung cell subgroup [9]. The subsequent viral infection can induce innate and adaptive immune responses leading to severe consequences [10]. Severe cases of COVID-19 exhibit a high concentration of activated immune cells,excessive production of inflammatory cytokines and chemical mediators, leading to uncontrolled innate and impaired adaptive immune responses [11]. SARS-CoV-2 can cause lung and systemic inflammation leading to ARDS or MODS, further aggravating the patient’s condition and increasing COVID-19 deaths [12–14].

ARDS, as a severe acute lung injury, is characterized by excessive and uncontrolled systemic inflammation and multiple organ microvascular endothelial cell damage. It was not just limited to pulmonary inflammation but can also caused systemic inflammation in severe cases [15]. It is further demonstrated by studies conducted on intensive care unit (ICU) patients in January 2020. The results showed that when compared to their non-ICU counterparts, COVID-19 patients in the ICU (n = 41) exhibited higher serum levels of interleukin-2, interleukin-7, interleukin-10, monocyte chemotactic protein 1, tumor necrosis factor-alpha [16, 17]. The increase of proinflammatory factors in the lung can lead to a local activation and damage of the pulmonary alveolus and its proximal vascular endothelial cells [9]. Moreover, SARS-CoV-2 can directly damage lung epithelial cells, therefore destroying their tight junction and changing their morphology [18]. Autopsy performed on COVID-19 victims showed diffuse alveolar damages due to acute interstitial pneumonia, microvascular fibrin deposition, perivascular T lymphocytes infiltration, and intravascular neutrophil retention [19, 20]. Excessive neutrophil retention can lead to bronchial obstruction, endothelial damage, and abnormal gas exchange, therefore promoting the occurrence of ARDS [21]. Additionally,changes such as neutrophil activation or delayed apoptosis and clearance are closely associated with ARDS [22]. Indeed, neutrophils can produce granin and chromatin, when associated with the pathogen, they can form neutrophil-derived extracellular traps (NETs), providing a pathway for platelet aggregation and fibrin deposition, significantly contributing to the formation of venous thrombosis [23]. The number of circulating neutrophils was increased in COVID-19 patients with severe disease, and NETs concentrations in plasma, tracheal inhalation, and lung tissue were increased in COVID-19 patients at autopsy. Covid-19 virus activates neutrophils to release NETs through the ACE2/TMPRSS2 pathway, promoting lung epithelial cell apoptosis and lung injury.One study showed that neutrophils in the lungs of patients with COVID-19 may enhance thrombosis by activating platelets[24]. COVID-19 can also induce hypoxemia, activation of the coagulation cascade, endothelial cell destruction, and activation of tissue factor expression, leading to extensive microvascular thrombosis [9]. Indeed, a preliminary report has shown that the majority of fatalities are due to a combination of hypoxic respiratory failure and acute respiratory distress [25].

A study on 91 COVID-19 related deaths performed by the people’s Hospital of Wuhan University has indicated that 73 subjects suffered from ARDS and 14 of MODS [26]. A study of autopsy reports from 23 COVID-19 cases (United States of America) also confirmed that thrombosis often occurred in small arteries of such patients, including 5 instances of pulmonary embolism coupled with pulmonary infarction or hemorrhage [20]. Another investigation of autopsy reports from 10 elderly African American patients found that the principal cause of death was thrombosis in small pulmonary vessels and capillaries [27]. A separate autopsy study of 7 patients who died from COVID-19 confirmed that, COVID-19 patients also have a higher incidence of microthrombi in the alveolar-capillary [19]. In conclusion, uncontrolled systemic inflammatory response and multi-organ microvascular endothelial damage is the pathological basis for death of COVID-19 patients.

## Elderly COVID-19 patients with cardiovascular disease have a higher risk of uncontrolled systemic inflammatory response and multi-organ microvascular endothelial damage

A previous study on 83 COVID-19 subjects showed a higher mortality rate of those with CVD, with severe lung tissue damages [28]. They are more likely to develop respiratory failure and develop into severe cases [29]. In a retrospective study of 200 Chinese patients with COVID-19, the authors showed that COVID-19 patients in the ICU are usually older and more prone to dyspnea and other complications such as ARDS [30]. More than half (80.5 %) of COVID-19 related deaths are over 60 years old, with a median age of 72.5 years old. Most deceased patients had a preexisting condition (76.8 %), with the top three comorbidities being hypertension, heart disease, and diabetes [31]. Another study on 100 cases of COVID-19 deaths (China) has demonstrated that elderly patients with chronic diseases, especially cardiovascular diseases and diabetes, accounted for the majority of COVID-19 related deaths. The main and most commonly seen direct cause of death among this subgroup are ARDS and MODS [32]. In the autopsy study of 7 COVID-2019 patients aged 68 to 80 years, indicating extensive thrombosis with microvascular lesions [19]. Additionally, autopsies of 23 COVID-19 cases in the United States of America also confirmed that thrombosis often occurred in small arteries of infected chronic heart disease patients [20].

## Possible mechanisms leading to high mortality in elderly COVID-19 patients with cardiovascular disease

### COVID-19 aggravated the imbalance of immune function of the elderly

Previous findings have also indicated that compared with younger subjects, the number of T lymphocytes and B lymphocytes, macrophages, granulocytes, periarterial lymphatic sheath proliferative cells, marginal zone, and lymphatic follicles were significantly decreased in the elderly. On the other hand, with the growth of age, the maturity rate of newly generated T lymphocytes in the body decreases, the number of aging macrophages, mast cells and apoptotic cells is increasing [33, 34]. The increase in the Th17/Treg cell ratio of the elderly may cause changes in the basic levels of pro-inflammatory cells [35]. Aging macrophages and granulocytes show enhanced pro-inflammatory state and are prone to transition to inflammatory phenotype,secret a large number of atypical pro-inflammatory cytokines, resulting in impaired phagocytosis, migration and clearance; further resulting in persistent inflammatory state of lung tissue, which is incapable of fighting respiratory pathogens and prone to acute lung injury [36].

Experimental studies in animals found that with the change of age, the function of mouse T lymphoid follicular regulatory cells was inhibited, the function of T lymphoid follicular helper cells was impaired, and macrophages had inherent defects in anti-inflammatory response [37, 38]. These changes not only resulted in a lower response to lymphocytes to activation and proliferation signals [39], but also reduced sensitivity to injury-induced apoptosis, leading to the accumulation of dysfunctional cells [40]. In this regard, studies have found that oxidative stress and chronic antigen load decrease the susceptibility of lymphocytes to injurious cell death with the increase of age, and enhance the pro-inflammatory state, leading to uncontrolled immune inflammatory response [35].

The storm of inflammatory mediators caused by COVID-19 infection can impair the innate and adaptive immune responses [41]. However,due to the tissue damage caused by this, the human body lacks effective immune memory, so it cannot effectively eliminate the virus [42]. SARS-CoV-2 triggered significant proinflammatory cytokine/chemokine expression of macrophages [43]. Plasmacytoid dendritic cells(pDCs )decreased in severe patients infected with SARS-CoV-2, the response to promoting macrophage clearance was impaired. The number of proinflammatory monocyte derived dendritic cells increased significantly. SARS-CoV-2 can infect pulmonary interstitial dendritic cells through ACE2 and express of dendritic cell-specific intracellular adhesion molecule-grabbing nonintegrin (DC-SIGN), which significantly promotes the binding of the virus to the receptor. The elderly (> 60 years old) reduced the number of pDCs, the level of proinflammatory cytokines secret by dendritic cells increased, the expression of DC-SIGN gene was higher in dendritic cells infected with SARS-CoV-2. So older people are more severely infected with SARS-CoV-2 [44]. It has been found that the total number of circulating lymphocytes, CD4 + T cells, CD8 + T cells and B lymphocytes are decreased in patients with ARDS or death of COVID-19 [42, 45]. The change in the number of cells was more pronounced in older patients. A possible explanation is COVID-19 causes spleen and lymph node atrophy, as well as a depletion of lymphocytic organ cells [46]. Another possible reason might be the release of cytokines (especially interleukin-6 and tumor necrosis factor-alpha) [45]. It causes dendritic cells to recognize viral antigens from the alveoli, therefore leading to a massive CD4 + T and CD8 + T cells recruitment to lung tissues [42]. While the activation of CD8 + T cells is delayed or damaged [45]. This state leads to ineffective activation of CD8 + T cells and natural killer T lymphocytes, which in turn prevents the body from producing enough antibodies to clear the virus effectively. These mediators, in turn, further stimulate macrophage activation, triggering inflammatory storms. Prolonged inflammatory storms can destroy lung epithelial cells, leading to severe lung damage and even multiple organ failure [42].

### The impact of COVID-19 on cardiovascular disease

Risk factors such as hypertension and hyperlipidemia stimulate the endothelial cells from anti-inflammatory to pro-inflammatory phenotypes causing endothelial cell damage [47, 48]. Endothelial injury overexpressed vascular cell adhesion molecule-1, intercellular adhesion molecule-1,recruited T lymphocytes, macrophages and dendritic cells. Dendritic cells drift into the lymph nodes to the focal drainage lymph nodes and/or the spleen, where primitive T cells develop into effector T cells and re-enter the bloodstream, resulting in atherosclerotic lesion instability [49]. This is a failed immune response that cannot solve inflammation and repair endothelial injury [50]. Most of the intima was proinflammatory monocyte phenotype dendritic cells. In atherosclerotic plaques in patients with coronary heart disease, the number of plasmacytoid DCs (pDCs) with anti-AS are reduced [51, 52]. In severe patients infected with SARS-CoV-2, pDCs decreased, and the secretion of inflammatory cytokines in monocyte derived dendritic cells and macrophages increased [53, 54], which promoted atherosclerotic plaque inflammation and led to plaque instability. COVID-19 infects vascular endothelial cells, microvascular pericytes by ACE2 receptors, aggravating endothelial injury to patients with CVD [55]. Atherosclerotic plaque is accompanied by chronic inflammation [56, 57]. Inflammatory cytokines such as C-reactive protein, erythrocyte sedimentation rate in blood of patients with COVID-19 infection with CVD were significantly higher than those without CVD [28]. A large number of inflammatory cytokines in the blood circulation can aggravate endothelial injury, activate platelets, aggravate blood coagulation state, then cause thrombosis among coronary blood vessels and microvascular are similar to acute coronary syndromes [58–60]. In summary, patients with COVID-19 infection with CVD have more severe inflammatory factors, increase vascular, microvascular endothelial injury and thrombosis. At the same time, through autopsy of COVID-19 infected patients, extensive infiltration of neutrophils, macrophages and CD4 + T lymphocytes were found in their cardiomyocytes [55]. Most reports report that COVID-19 caused immune inflammation in the interstitial of the heart, while a few report that COVID-19 virus directly infiltrated the myocardium causing cardiac function damage, similar to viral myocarditis [61–63]. A large number of inflammatory cytokines caused by COVID-19 infection leading to microvascular damage and thrombosis, myocardial damage is one of the main causes of death [64, 65].

A hyperinflammatory syndrome reminiscent of toxic shock syndrome (TSS) is observed in severe COVID-19 patients, including Pediatric Inflammatory Multisystem Syndrome temporally related to COVID-19 (PIMS-TS).TSS are characterized, by fever, systemicinflammation, abdominal pain and cardiac involvement [66, 67]. TSS is superantigen by bacteria including Staphylococcus aureus and Streptococcus pyogenes. The interaction of superantigen with specific TCR Vβregions and may form a ternary complex about major histocompatibility complex class II(MHCII), activates clonal expression of 20–30 % of host T cells, release a large number of cytokines led to multiple organ tissue damage [67], cardiac involvement is the most serious. After this proliferative phase, T cells enter a state of deep depletion, or even cell death [68].

A study showed that the binding epitope on S harbors a sequence motif unique to SARS-CoV-2, which is highly similar in both sequence and structure to the bacterial superantigen staphylococcal enterotoxin B (SEB)and may directly bind T cell receptors. They further report a skewed T cell receptor repertoire in COVID-19 patients with severe hyperinflammation, in support of such a superantigenic effect [66, 69]. It has been suggested that Intravenous immunoglobulin (IVIG) can block in-vitro T cells activation by Staphylococcal and streptococcal superantigens. IVIG used for TSS had been shown to be effective for PIMS-TS [67]. A study found that CD4 + T cells and CD8 + T cells were significantly reduced by in patients with COVID-19 in ICU in Wuhan [70]. Activated phenotypes of CD4 + T cells and CD8 + T cells were found in patients with COVID-19, and markers of T cell exhaustion both in lung and circulating were upregulated [71]. It is inferred that in COVID-19 patients, there are a large number of T cells activations and subsequent exhausted, so the immune response cannot be formed effectively.

Streptococcus is the cause of rheumatic fever, rheumatic fever causes rheumatic heart disease is mainly streptococcal antigen and human body organization (streptococcal M protein and myocardial myoplasma globulin, bacteria wall polysaccharide and heart valve) exist cross antigen. Antibody produced inflammatory cytokines activate heart valve endothelial cells and express vascular cell adhesion factor 1. Subsequently, CD4 + T cells, CD8 + T cells infiltrate and damage the heart valve, trigger rheumatic heart disease that can lead to heart failure, infection and severe arrhythmias [72, 73]. SARS-CoV-2 is not acute rheumatic fever, although the presence of positive group A Streptococcus testing, antistreptolysin O and antiDNAse B titers ultimately remained negative. There is no obvious heart valve inflammation, but myocardial involved [74].

Rheumatic disease receiving immunomodulatory therapy was not associated with the severity of COVID-19 [69]. Poor control of rheumatic activity and higher inflammatory markers (such as C-reactive protein, serum ferritin) were associated with COVID-19 severity [75]. 80 % of individuals over 12 years old harbor anti-SEB antibodies and protective SEB titers fall in older adults after 70 years old. In Europe, it was found that the mutation D839Y of SARS-CoV-2 enhances the binding affinity with the SARS-CoV-2 spike (S) glycoprotein to the TCR. With a history of SARS-CoV-2 exposure, the antibody mediated immunity was enhanced after re-exposure, which may lead to uncontrolled infection and inflammation [66]. This paper proposes a hypothesis, older people infected COVID-19 who infection of staphylococcus and streptococcus during childhood, will be easier progressing to severe illness and death. The mechanism is the excessive activation of immune inflammatory cytokines lead to multiple organ failure, including myocardial injury.

## Conclusions

SARS-CoV-2 is a novel coronavirus with significant implications for the human immune system. Its effects are even more apparent to elderly patients with CVD due to their aging immune system. COVID-19 infection in this subgroup often aggravated the existing imbalance within their innate and adaptive immune responses, caused lymphocyte depletion and an uncontrollable inflammatory response due to cytokine storm. It’s an inadequate sustained immune response, a lack of effective immune memory, an inability to clear the virus. It caused injury to the lung epithelium and microvascular endothelium throughout the body. Therefore leading to ARDS, MODS and microvascular thrombosis, with the outcome being high mortality among elderly COVID-19 patients with CVD.

## Data Availability

Not applicable.

## **Authors’ information**

No further information.

## References

[1] Fei Zhou T, Yu R, Du,et al.Clinical course and risk factors for mortality of adult inpatients with COVID-19 in Wuhan, China: a retrospective cohort study.Lancet 2020; 395: 1054-62. 10.1016/S0140-6736(20)30566-3 PMC7270627 32171076

[2] Zhang J (2020). Shimin Lu,Xiaoli Wang,et al.Do underlying cardiovascular diseases have any impact on hospitalised patients with. COVID-19? Heart.

[3] Li M, Wang YDong,H (2020). Cardiovascular disease potentially contributes to the progression and poor prognosis of COVID-19.Nutrition. Metabolism Cardiovascular Diseases.

[4] Wei Pan J, Zhang M, Wang (2020). Clinical Features of COVID-19 in Patients With Essential Hypertension and the Impacts of Renin-angiotensin-aldosterone System Inhibitors on the Prognosis of COVID-19. PatientsHypertension.

[5] Carolin Edler AS, Schröder M, Aepfelbacher (2020). Dying with SARS-CoV-2 infection-an autopsy study of the first consecutive 80 cases in Hamburg. GermanyInt J Legal Med.

[6] Sun Y-J, Feng Y-J, Chen J, Li B (2020). Zhong-Cheng Luo,Pei-Xi Wang.Clinical Features of Fatalities in Patients With COVID-19. Disaster Med Public Health Prep.

[7] Zangrillo A, Beretta L, Scandroglio AM,et al.Characteristics, treatment, outcomes and cause of death of invasively ventilated patients with COVID-19 ARDS in Milan, Italy. Crit Care Resusc 2020 Apr 23. 10.1016/S1441-2772(23)00387-3 PMC10692521 32900326

[8] Jolanda Sabatino S, De Rosa GD, Salvo C, Indolfi. Impact of cardiovascular risk profile on COVID-19 outcome. A meta-analysis. PLoS One 2020. Accepted July 21, 2020. 10.1371/journal.pone.0237131 PMC7428172 32797054

[9] Dennis McGonagle, James S, O’Donnell K, Sharif (2020). Paul Emery, Charles Bridgewood.Immune mechanisms of pulmonary intravascular coagulopathy in COVID-19 pneumonia. Lancet Rheumatol.

[10] Adriaan H, de Wilde EJ, Snijder M, Kikkert, Martijn J van. HemertHost Factors in Coronavirus ReplicationCurr Top Microbiol Immunol. 2018;419:1–42. 10.1007/82_2017_25 PMC7119980 28643204

[11] Biying Hu, Huang S. Lianghong Yin.The cytokine storm and COVID- 19.J Med Virol 2020;Jun 27. 10.1002/jmv.26232 PMC7361342 32592501

[12] Qing Ye B, Wang (2020). Jianhua Mao.The pathogenesis and treatment of the Cytokine Storm’ in COVID-19. J Infect.

[13] Tao Chen D, Wu H, Chen,et al. Clinical characteristics of 113 deceased patients with coronavirus disease 2019: retrospective study.BMJ 2020;368:1091. 10.1136/bmj.m1091 PMC7190011 32217556

[14] Peng YD, Meng K, Guan HQ (2020). Clinical characteristics and outcomes of 112 cardiovascular disease patients infected by SARS-CoV-2. Zhonghua Xin Xue Guan Bing Za Zhi.

[15] Sun G, Qian G (1997). J Xu.Systemic inflammatory response syndrome and acute lung injury, multiple organ dysfunction syndrome.Zhonghua Jie He He Hu Xi. Za Zhi.

[16] Chaolin Huang,Yeming Wang,Xingwang Li,et al.Clinical features of patients infected with 2019 novel coronavirus in Wuhan, China.Lancet 2020;395:497–506. 10.1016/S0140-6736(20)30183-5 PMC7159299 31986264

[17] Brit Long,William J, Brady A, Koyfman (2020). Michael Gottlieb.Cardiovascular complications in COVID-19.Am. J Emerg Med.

[18] Widagdo W, Ayudhya SSooksawasdiN, Gadissa B, Hundie (2019). Bart L Haagmans.Host Determinants of MERS-CoV. Transmission PathogenesisViruses.

[19] Maximilian Ackermann SE, Verleden M, Kuehnel (2020). Pulmonary Vascular Endothelialitis, Thrombosis, and Angiogenesis in Covid-19.N Engl. J Med.

[20] Buja LM, Wolf DA, Zhao B (2020). The emerging spectrum of cardiopulmonary pathology of the coronavirus disease 2019 (COVID-19): Report of 3 autopsies from Houston, Texas, and review of autopsy findings from other United States cities. Cardiovasc Pathol.

[21] Wong JJuM, Leong JY, Lee JH (2019). Salvatore Albani, Joo Guan Yeo.Insights into the immuno-pathogenesis of acute respiratory distress syndrome. Ann Transl Med.

[22] Arlette Vassallo AJ, Wood J, Subburayalu C, Summers, Edwin R, Chilvers (2019). The counter-intuitive role of the neutrophil in the acute respiratory distress syndrome. Br Med Bull.

[23] Saghazadeh A (2016). Nima Rezaei.Inflammation as a cause of venous thromboembolism. Crit Rev Oncol Hematol.

[24] Veras FP, Pontelli MC (2020). Camila Meirelles Silva,et al.SARS-CoV-2 -triggered neutrophil extracellular traps mediate COVID-19 pathology. J Exp Med.

[25] Keshava Rajagopal SP, Keller B, Akkanti,et al. Advanced Pulmonary and Cardiac Support of COVID-19 Patients: Emerging Recommendations From ASAIO-A “Living Working Document”. ASAIO J. 2020;66:588–98. 10.1097/MAT.0000000000001180 PMC7217129 32358232

[26] Yang F, Shi S, Zhu J, Shi J, Dai K, Chen X,et al.Analysis of 92 deceased patients with COVID-19.J Med Virol 2020; Apr 15. 10.1002/jmv.25891 PMC7262332 32293741

[27] Fox SE, Akmatbekov A, Harbert JL, Li G, Brown JQ. Richard S Vander Heide.Pulmonary and cardiac pathology in African American patients with COVID-19: an autopsy series from New Orleans. Lancet Respir Med. 2020;8:681–6. 10.1016/S2213-2600(20)30243-5 PMC7255143 32473124

[28] Li M (2020). Yalan Dong,Haijun Wang,et al.Cardiovascular disease potentially contributes to the progression and poor prognosis of COVID-19.Nutr Metab. Cardiovasc Dis.

[29] Akanksha N, Thakkar I, Tea,Mouaz H, Al-Mallah (2020). Cardiovascular Implications of COVID-19 Infections.Methodist Debakey. Cardiovasc J.

[30] Yang L, Liu J, Zhang R (2020). Epidemiological and clinical features of 200 hospitalized patients with corona virus disease 2019 outside Wuhan, China: A descriptive study. J Clin Virol.

[31] Bicheng Zhang,Xiaoyang Zhou,Yanru Qiu,et al.et al.Clinical characteristics of 82 cases of death from COVID-19.PLoS One 2020;15:0235458. 10.1371/journal.pone.0235458 PMC7347130 32645044

[32] Li Ya-JunSun,Yi-JinFeng,JingChen,Bo, Luo Z-C. Pei-Xi Wang.Clinical Features of Fatalities in Patients With COVID-19.Disaster Med Public Health Prep 2020;1–3. 10.1017/dmp.2020.235 PMC7443557 32665052

[33] Graham Pawelec Y, Barnett. Ros Forsey,et al.T cells and aging,January 2002 update.Front Biosci 2002;7:1056–183. 10.2741/a831 11991846

[34] Nesma I, El-Naseery, Hanaa SE, Mousa AE, Noreldin AH, El-Far (2020). Yaser H A Elewa.Aging-associated immunosenescence via alterations in splenic immune cell populations in rat. Life Sci.

[35] Ewa Sikora.Activation-induced and damage-induced cell death in aging human T cells.Mech Ageing Dev 2015;151:85–92. 10.1016/j.mad.2015.03.011 25843236

[36] Christina Brandenberger KM, Kling M, Vital (2018). Mühlfeld Christian.The Role of Pulmonary and Systemic Immunosenescence in Acute Lung Injury. Aging Dis.

[37] Goutham Pattabiraman K, Palasiewicz JP, Galvin (2017). David S Ucker.Aging-associated dysregulation of homeostatic immune response termination (and not initiation). Aging Cell.

[38] D Zharhary.Age-related changes in the capability of the bone marrow to generate B cells.J Immunol 1988;141:1863–9. 3262642

[39] Ginaldi L, De Martinis M, D’Ostilio A, Marini L, Loreto MF, Corsi MP (2000). D Quaglino.Cell proliferation and apoptosis in the immune system in the elderly. Immunol Res.

[40] Maria Teresa Ventura,Marco Casciaro,Sebastiano Gangemi,Rosalba Buquicchio.Immunosenescence in aging: between immune cells depletion and cytokines up-regulation.Clin Mol Allergy 2017;15:21. 10.1186/s12948-017-0077-0 PMC5731094 29259496

[41] Sophie Hue,Asma Beldi-Ferchiou,Inès Bendib,et al.Uncontrolled Innate and Impaired Adaptive Immune Responses in Patients with COVID-19 ARDS.Am J Respir Crit Care Med 2020; Aug 31. 10.1164/rccm.202005-1885OC PMC7706149 32866033

[42] PeterP L (2020). Alice Blet,David Smyth,Hongliang Li.The Science Underlying COVID-19: Implications for the Cardiovascular. SystemCirculation.

[43] Chu DYang,Hin, Hou Y (2020). Attenuated Interferon and Proinflammatory Response in SARS-CoV-2 -Infected Human Dendritic Cells Is Associated With Viral Antagonism of STAT1 Phosphorylation. J Infect Dis.

[44] Borges RC, Hohmann MS. Sergio Marques Borghi.Dendritic cells in COVID-19 immunopathogenesis: insights for a possible role in determining disease outcome.Int Rev Immunol 2020;1–18. 10.1080/08830185.2020.1844195 33191813

[45] Hue S, Beldi-Ferchiou A. Inès Bendib,et al.Uncontrolled Innate and Impaired Adaptive Immune Responses in Patients with COVID-19 ARDS.Am J Respir Crit Care Med 2020. 10.1164/rccm.202005-1885OC PMC7706149 32866033

[46] Roshanravan N, Seif F, Ostadrahimi A, Pouraghaei M (2020). Samad Ghaffari.Targeting Cytokine Storm to Manage Patients with COVID-19: A Mini-Review. Arch Med Res.

[47] Ying Shao J, Saredy WY, Yang (2020). Vascular Endothelial Cells and Innate Immunity. Arterioscler Thromb Vasc Biol.

[48] Ia, Pantsulaia (2016). Wojciech Michal Ciszewski, Jolanta Niewiarowska.Senescent endothelial cells: Potential modulators of immunosenescence and ageing. Ageing Res Rev.

[49] Harald Mangge,Gunter Almer.Immune-Mediated Inflammation in Vulnerable Atherosclerotic Plaques.Molecules 2019;24:3072. 10.3390/molecules24173072 PMC6749340 31450823

[50] Daria V, Ilatovskaya,Ganesh V, Halade,Kristine Y (2019). DeLeon-Pennell.Adaptive immunity-driven inflammation and cardiovascular disease. Am J Physiol Heart Circ Physiol.

[51] Dieterlen M-T, John K, Reichenspurner H, Mohr FW, Markus J (2016). Barten.Dendritic Cells and Their Role in Cardiovascular Diseases: A View on Human Studies. J Immunol Res.

[52] Christ A, Temmerman,Bart Legein,Mat L, Daemen,Erik JAP (2013). A L Biessen.Dendritic cells in cardiovascular diseases: epiphenomenon, contributor. or therapeutic opportunityCirculation.

[53] To RZhou,KKai-Wang, Wong Y-C (2020). Acute SARS-CoV-2 Infection Impairs Dendritic Cell and. T Cell ResponsesImmunity.

[54] Zheng J, Wang Y, Li K, Meyerholz DK, Allamargot C. Stanley Perlman.SARS-CoV-2 -induced immune activation and death of monocyte-derived human macrophages and dendritic cells.J Infect Dis 2020;jiaa753. 10.1093/infdis/jiaa753 PMC7799009 33277988

[55] Jung F, Krüger-Genge A, Franke RP, Hufert F (2020). J-H Küpper.COVID-19 and the endothelium. Clin Hemorheol Microcirc.

[56] Vanessa Frodermann,Matthias Nahrendorf.Macrophages and Cardiovascular Health.Physiol Rev 2018;98:2523–69. 10.1152/physrev.00068.2017 PMC6442921 30156496

[57] Wolf D (2019). Klaus Ley.Immunity and Inflammation in Atherosclerosis. Circ Res.

[58] Jean M, Connors,Jerrold H (2020). Levy.COVID-19 and its implications for. thrombosis anticoagulationBlood.

[59] James D (2020). McFadyen,Hannah Stevens, Karlheinz Peter.The Emerging Threat of (Micro)Thrombosis in COVID-19 and Its Therapeutic Implications. Circ Res.

[60] Meaghan E (2020). Colling,Yogendra Kanthi.COVID-19-associated coagulopathy: An exploration of mechanisms. Vasc Med.

[61] Ashar Pirzada, Ahmed T, Mokhtar AD. Moeller.COVID-19 and Myocarditis: What Do We Know So Far?CJC Open 2020;2:278–85. 10.1016/j.cjco.2020.05.005 PMC7254016 32691024

[62] Khalid Sawalha M, Abozenah AJ, Kadado,et al.Systematic review of COVID-19 related myocarditis: Insights on management and outcome.Cardiovasc Revasc Med 2020;Aug 18. 10.1016/j.carrev.2020.08.028 PMC7434380 32847728

[63] Pramod Theetha Kariyanna,Bayu Sutarjono,Ekjot Grewal,et al.A Systematic Review of COVID-19 and Myocarditis.Am J Med Case Rep 2020;8:299–305.

[64] Anuradha Lala KW, Johnson JL, Januzzi (2020). Prevalence and Impact of Myocardial Injury in Patients Hospitalized With COVID-19 Infection. J Am Coll Cardiol.

[65] Mohit D, Gupta MP, Girish,Geetika (2020). Yadav,Abhishek Shankar, Rakesh Yadav.Coronavirus disease 2019 and the cardiovascular system: Impacts and implications. Indian Heart J.

[66] Cheng MH, Zhang S, Rebecca A, Porritt (2020). Superantigenic character of an insert unique to SARS-CoV-2 spike supported by skewed TCR repertoire in patients with hyperinflammation. Proc Natl Acad Sci U S A.

[67] Buonsenso D, Riitano F (2020). Piero Valentini.Pediatric Inflammatory Multisystem Syndrome Temporally Related With SARS-CoV-2 : Immunological Similarities With Acute Rheumatic Fever and Toxic Shock Syndrome. Front Pediatr.

[68] Goran Abdurrahman F, Schmiedeke C, Bachert BM, Bröker. Silva Holtfreter.Allergy-A New Role for T Cell Superantigens of Staphylococcus aureus?Toxins (Basel)2020;12(3):176. 10.3390/toxins12030176 PMC7150838 32178378

[69] Fatih Haşlak M, Yıldız A, Adrovic K, Barut (2020). Özgür Kasapçopur.Childhood Rheumatic Diseases and COVID-19 Pandemic: An Intriguing Linkage and a New Horizon. Balkan Med J.

[70] Choudhury I, Han H, Manthani K, Gandhi S (2020). Rameshchandra Dabhi.COVID-19 as a Possible Cause of Functional Exhaustion of CD4 and CD8 T-cells and Persistent Cause of Methicillin-Sensitive. Staphylococcus aureus BacteremiaCureus.

[71] Martin H, Stradner C, Dejaco J, Zwerina RD, Fritsch-Stork (2020). Rheumatic Musculoskeletal Diseases and COVID-19 A Review of the First 6 Months of the Pandemic. Front Med (Lausanne).

[72] Smoot LM, McCormick JK, Smoot JC (2002). Chara cterization of two novel pyrogenic toxin superantigens made by an acute rheumatic fever clone of Strep tococcus pyogenes associated with multiple disease out breaks. Infect Immun.

[73] CunninghamMW.Autoimmunity and molecular mimicry in the pathogenesis of poststreptococcal heart disease.Front Biosci 2003;8:533 – 43. 10.2741/1067 12700052

[74] Daniel Vari JM, Miller N, Rellosa S, Srivastava M, Frizzola (2020). Deepika Thacker.Severe cardiac dysfunction in a patient with multisystem inflammatory syndrome in children associated with COVID-19: Retrospective diagnosis of a puzzling presentation. A case report. Prog Pediatr Cardiol.

[75] Sieiro Santos C, Moriano Morales C, Díez Álvarez E, C Álvarez Castro, A López Robles, T Perez Sandoval.Determinants of COVID-19 disease severity in patients with underlying rheumatic disease.Clin Rheumatol 2020;39(9):2789-96. 10.1007/s10067-020-05301-2 PMC7383119 32720259

